# Lipocalin 2 regulates iron homeostasis, neuroinflammation, and insulin resistance in the brains of patients with dementia: Evidence from the current literature

**DOI:** 10.1111/cns.13653

**Published:** 2021-05-04

**Authors:** Daejin Lim, Jae‐ho Jeong, Juhyun Song

**Affiliations:** ^1^ Department of Microbiology Chonnam National University Medical School Gwangju Korea; ^2^ Department of Anatomy Chonnam National University Medical School Chonnam National University Gwangju Korea

**Keywords:** dementia, insulin resistance, iron homeostasis, lipocalin 2 (LCN2), neuroinflammation

## Abstract

Dementia accompanied by memory loss is considered one of the most common neurodegenerative diseases worldwide, and its prevalence is gradually increasing. Known risk factors for dementia include genetic background, certain lifestyle and dietary patterns, smoking, iron overload, insulin resistance, and impaired glucose metabolism in the brain. Here, we review recent evidence on the regulatory role of lipocalin 2 (LCN2) in dementia from various perspectives. LCN2 is a neutrophil gelatinase‐associated protein that influences diverse cellular processes, including the immune system, iron homeostasis, lipid metabolism, and inflammatory responses. Although its functions within the peripheral system are most widely recognized, recent findings have revealed links between LCN2 and central nervous system diseases, as well as novel roles for LCN2 in neurons and glia. Furthermore, LCN2 may modulate diverse pathological mechanisms involved in dementia. Taken together, LCN2 is a promising therapeutic target with which to address the neuropathology of dementia.

## Graphical abstract

The role of LCN2 in neuronal cell damage in the brain of dementia patients.

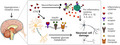

## 1 INTRODUCTION

Dementia is a common neurodegenerative disorder with a markedly increasing worldwide prevalence.^1^, ^2^, ^3^ The term dementia is used as a general, umbrella term for vascular dementia (VD), Lewy body dementias (LBD), frontotemporal dementia (FTD), and Alzheimer's disease (AD). The major neurological features of dementia are cognitive impairment and neuroinflammation.^4^, ^5^ Chronic neuroinflammation, neuronal loss, oxidative stress, amyloid beta accumulation, and impaired synaptic plasticity lead to memory loss.^6^, ^7^ Furthermore, a chronic inflammatory state gives rise to the activation of the tau kinase, increasing the formation of intracellular neurofibrillary tangles, leading to synapse dysfunction.^6^

Multiple risk factors for dementia have been reported, including genomic mutations, impaired lipid metabolism, and impaired glucose metabolism.^8^ Furthermore, several studies have reported that changes in the metabolic state, including blunt insulin sensitivity and hyperglycemia, are directly linked to the onset and development of dementia, especially memory deficits.^6^, ^9^ Thus, a complete understanding of the risk factors for dementia will allow the underlying mechanisms to be characterized and targeted for therapeutic approaches.

Lipocalin 2 (LCN2), also known as neutrophil gelatinase‐associated lipocalin (NGAL), functions in the regulation of the immune system and in inflammatory processes.^10^, ^11^ LCN2 is an antibacterial protein that acts by sequestering iron during bacterial infection and has recently been reported to be involved in various pathophysiological conditions in various organs and tissues, including the heart, lung, liver, kidney, and brain.^12^, ^13^

Recent studies have suggested that LCN2 modulates cellular activity in the central nervous system (CNS) and peripheral nervous system through the activation of glia, the control of iron accumulation, and the regulation of neuroinflammation, which is defined as an inflammatory response and neurotransmitter secretion within the CNS.^14^, ^15^, ^16^, ^17^ Additionally, LCN2 can cross the blood‐brain barrier (BBB) by binding with the melanocortin 4 receptor (MC4R) in hypothalamic neurons.^18^ Another study demonstrated that cancer cells are growing in the LCN2/SLC22A17 system in the leptomeningeal metastasis mouse model.^19^

Lipocalin 2 likely regulates cell differentiation by modulating iron transport and cell death signaling, such as controlling the activation of the nuclear factor kappa‐light‐chain‐enhancer of activated B cells (NF‐κB). LCN2 ultimately controls neuroinflammation by modulating the production of cytokines, including interleukin‐1 beta (IL‐1β), tumor necrosis factor‐α (TNF‐α), and interleukin‐6 (IL‐6) secreted from glia.^15^ In contrast, LCN2 also has neuroprotective functions in the brain by suppressing the secretion of pro‐inflammatory cytokines.^18^

In addition, modulating LCN2 may be useful for preventing BBB disruptions and white matter atrophy that lead to neurological diseases, including stroke and dementia.^14^, ^20^ Furthermore, LCN2 is involved in the accumulation of iron that occurs within brain neurons of patients with dementia and is related to cognitive dysfunction.^21^ LCN2 can also decrease synaptic plasticity through its involvement in controlling active mobility through iron‐dependent signaling.^22^ Moreover, LCN2 is also strongly related to an impaired metabolic state, such as impaired glucose metabolism, thus leading to cognitive decline.^23^, ^24^ Finally, LCN2‐deficient mice display increased insulin resistance under hyperglycemia and heightened blood glucose levels compared to control mice.^23^

Here, we review the evidence for the involvement of LCN2 in dementia, focusing on neuroinflammation, iron accumulation, and metabolic alterations. These findings highlight the importance of the role of LCN2 in the CNS and suggest that LCN2 is key to modulating the neuropathology of dementia.

## 2 DEMENTIA

Dementia is one of the most common disorders affecting elderly people, and the number of patients with dementia is gradually increasing globally.^1^ Dementia is generally classified into four subtypes and is used as a general term to describe a syndrome of progressive cognitive decline, including AD, LBD, FTD, and VD.^25^, ^26^ The main clinical pathologies of dementia are objective cognitive deficits, language impairments, and difficulties with executive function or judgment.^5^

Alzheimer's disease, the most common type of dementia, is characterized by the excessive accumulation of amyloid beta, neuronal damage, BBB disruption, tau hyperphosphorylation, and neurofibrillary tangles in several brain regions, including the hippocampus and the entorhinal cortex.^27^, ^28^, ^29^ Patients with AD show atrophy and degeneration of the cortex, as well as limbic and hippocampal regions, compared to healthy controls, which leads to memory loss.^30^, ^31^

Vascular dementia, the second most common type of dementia globally, represents approximately 20% of all dementia cases and results from neuronal damage caused by oxygen and glucose deprivation in various brain regions.^4^ VD, considered a multi‐infarct type of dementia, is influenced by lifestyle, dietary patterns, and vascular dysfunctions such as small vessel disease and lacunar infarctions.^4^, ^32^, ^33^

Lewy body dementias, the third most common type of dementia, results from the excessive accumulation of alpha‐synuclein protein, called Lewy bodies, in neurons.^34^ LBD leads to cognitive impairments with recurrent visual hallucinations, lethargy, and a decrease in attention, as well as parkinsonism.^34^, ^35^

Frontotemporal dementia is a general type of dementia that affects the frontal and temporal lobes and mainly occurs in younger individuals, as opposed to the generally older age of patients with AD. A hallmark of FTD is the high accumulation of neurofibrillary tangles in the frontal and parietal lobes.^36^ This type of dementia leads to a lack of judgment, inappropriate social behavior, and memory dysfunction.^37^ Importantly, the cognitive decline in dementia results from neuronal damage in the cerebral cortex, synaptic dysfunction, neuroinflammation, and impaired cerebral metabolism.^5^, ^38^, ^39^

Vascular dementia is caused by impaired vascular homeostasis, which aggravates cortical and hippocampal neuronal loss in the ischemic state, ultimately contributing to cognitive decline.^39^ In addition, changes in metabolic biomarkers contribute to impaired cerebral metabolism and ultimately damages neurons and glia by decreasing the supply of nutrients and oxygen.^38^

Numerous risk factors for dementia have been identified, including genetic predispositions, vascular damage, inflammation, comorbidities, as well as various lifestyle and physiological factors.^8^, ^40^ Furthermore, a number of studies have demonstrated a correlation between dementia and metabolic changes, such as insulin resistance (impaired intracellular insulin functioning) and glucose imbalance.^9^, ^41^ In fact, Bergantin et al^9^ found that dysregulation of cellular Ca^2+^ signaling represents an important risk factor, suggesting that it aggravates brain insulin resistance, resulting in memory loss.

Neuroinflammation aggravates neuronal loss and activates glia, ultimately leading to memory loss in dementia through excessive secretion of pro‐inflammatory cytokines and inflammatory mediators.^42^, ^43^ In the dementia brain, astrocytes and microglia are activated and secrete several inflammatory cytokines and neurotransmitters such as glutamate,^44^, ^45^ whereas neurons are damaged and show impaired synaptic plasticity against oxidative stress condition.^46^ Increased BBB permeability in dementia induces the infiltration of circulating immune cells such as T‐lymphocytes.^47^ A previous study found that brain insulin resistance, including impaired insulin receptor signaling and insulin‐like growth factor‐1 (IGF‐1) signaling, leads to cognitive dysfunction in dementia by aggravating neuronal cell death and synaptic dysfunction.^48^ Additionally, excessive iron accumulation observed in the caudate nucleus and putamen of VD patients contributes to neuronal synapse connectivity dysfunction and memory loss.^49^

Thus, the onset and progression of dementia are linked to many factors, which may contribute to the neuropathology observed in dementia. These previous studied have highlighted that there are few pathological differences between the different types of dementia.^8^ Further understanding of the potential risk factors for dementia is critical, as the onset of dementia results from diverse routes. Therefore, identifying specific risk factors for the onset and development of the disease is necessary for its prevention and treatment.

## 3 ROLES OF LCN2 IN DEMENTIA

Lipocalin 2 is a glycoprotein of the lipocalin superfamily comprised of 160–180 amino acids, which contributes to innate immunity and inflammatory responses.^10^, ^11^, ^50^ Two specific membrane receptors of LCN2 have been reported: megalin (known as LRP2, expressed by kidney epithelial cells) and 24p3R (known as solute carrier SLC22A17, expressed in various tissues, including the brain).^51^, ^52^ LCN2 can bind and transport both lipids and small hydrophobic molecules into the cell, and also control matrix metalloproteinases in blood vessels to mediate neurovascular remodeling.^53^, ^54^

Several studies have shown that LCN2 exerts multiple physiological cellular processes, such as inflammatory responses, and iron metabolism in the brain.^12^, ^15^, ^55^, ^56^, ^57^ LCN2 is an important modulator of innate responses, and exerts antibacterial and bacteriostatic effects through an interplay with the bacterial iron‐laden siderophore.^57^ LCN2 acts as a key controller of inflammation mediated through the toll‐like receptor 2 (TLR2) and TLR4 dependent pathways.^55^ Moreover, LCN2 blood levels are positively correlated with insulin resistance in females, as demonstrated in some clinical studies.^56^ Although the detailed mechanisms behind the role of LCN2 in these responses remain to be elucidated, the relevance between LCN2 and various cellular mechanisms should be highlighted in order to understand the roles of LCN2 in the brain.

In the CNS, increased LCN2 is observed in patients with neurodegenerative disorders such as AD and Parkinson disease, and ultimately aggravates the neuropathophysiology of such diseases.^50^, ^58^, ^59^ LCN2 is mainly produced in glia under oxidative stress in the brain ^50^ and can disrupt the BBB by boosting astrocyte and brain endothelial cell damage.^14^ One study demonstrated that LCN2‐deficient condition leads reduced cerebral cortex damage in a VD mouse model by decreasing neuroinflammation.^8^

Lipocalin 2 causes neuronal death, induces synaptic dysfunction, activates microglia, reduces white matter mass, and leads to microvasculature damage in the hippocampus by promoting the expression of vascular endothelial growth factor (VEGF).^8^

Several studies have shown that the level of LCN2 in the blood increases in patients with neurological diseases, including AD, mild cognitive impairment, and Parkinson's disease.^59^, ^60^ Mechanically, LCN2 regulates cellular responses by activating NF‐κB and hypoxia‐inducible factor‐1‐α (HIF‐1‐α) induction.^61^ Other in vivo and in vitro studies have reported that LCN2 is involved in the aggravation of pro‐inflammatory responses and the inhibition of neuroprotective cellular pathways in the brain by boosting the release of pro‐inflammatory cytokines such as TNF‐α.^58^, ^62^ Together, these findings suggest that LCN2 mediates the inflammatory state in the brain. Recently, LCN2 has emerged as a potential diagnostic marker for dementia,^63^ based on the observation of elevated LCN2 in the plasma of patients with dementia^64^ and in the cerebrospinal fluid (CSF) of patients with VD.^65^ In fact, a previous study demonstrated that LCN2 deficiency attenuates white matter damage and improves cognitive dysfunction in animal models of VD.^34^ Furthermore, in an in vitro model of AD, astrocytes secreted high amounts of LCN2, and elevated LCN2 ultimately boosted amyloid beta aggregation and amyloid beta‐induced cell death in astrocytes and endothelial cells.^58^, ^66^ Recent studies have also suggested that LCN2 is a strong and specific biomarker for VD.^38^, ^67^, ^68^, ^69^, ^70^

The relationship between the level of LCN2 and the pathological progression of dementia has been reported in previous studies. One study suggested that the CSF level of LCN2 is elevated in the brains of patients with VD.^50^ In addition, one cross‐sectional study showed that the plasma level of LCN2 was higher in patients with AD than in normal subjects.^71^

Based on these findings, we suspect that the levels of LCN2 may differ across the various models of dementia due to the difficulties in measuring the levels of LCN2 during the progress of dementia. Thus, although a strong relationship between neurodegenerative disease progression and LCN2 levels in the CNS and blood have been reported, clinicians should use caution when using LCN2 levels as a clinical indicator of neurodegenerative disease progression.

To summarize, LCN2 contributes to inflammatory responses and regulates various cell signaling pathways, affecting neurons and glia in the brains of patients with dementia. The fact that dementia is also linked to multiple mechanisms related to LCN2, such as inflammatory responses, iron metabolism, and cellular apoptosis, highlights the importance of the relationship between LCN2 and the neuropathology of dementia. Below, we review the roles of LCN2 in dementia from various perspectives.

In the CNS, iron is an essential biomolecule for cellular homeostasis, including DNA repair, DNA synthesis, oxidation, immune cell activation, synapse development, and cell division; it also acts as a neurotransmitter, such as in the development of neurodegenerative diseases.^72^, ^73^, ^74^ Specifically, iron is known to play a role in the development of neurodegenerative diseases by controlling monoamine neurotransmitters such as dopamine and serotonin and ultimately contributes to the regulation of cognitive functions, including emotional‐ and arousal‐related behaviors.^75^

Iron is also a co‐factor for multiple cellular physiological mechanisms, such as axonal myelination, oxidative mitochondrial metabolism (by promoting adenosine triphosphate [ATP] production), and the synthesis of neurotransmitters that depend on tyrosine hydroxylase.^76^, ^77^, ^78^ Iron is also a critical regulator of the oxidative stress response and is thus considered a redox‐active transition metal.^79^ Iron acts as an antioxidant regulator by catalyzing the formation of reactive oxygen species (ROS).^80^

Moreover, iron is an essential metal for the maintenance of energy consumption in the brain ‐ the most energy‐consuming metabolic organ in the body.^81^, ^82^ Furthermore, iron acts as a co‐factor in axonal myelination, mitochondrial function, and neurotransmission in the CNS.^83^

The main route of iron uptake begins with intestinal absorption in the gut, where dietary Fe^3+^ is reduced to Fe^2+^ by duodenal cytochrome B (DcytB), and divalent metal transporter 1 (DMT1) brings Fe^2+^ into the gut intestinal cells.^84^, ^85^ Thus, dietary intake of iron contributes to the balance of iron in the body and can offset iron deficiency.^86^ Intracellular iron transport requires iron transporters, such as DMT1 and transferrin receptor 1 (TfR1),^87^ as well as iron regulatory protein 1 (IRP1).^88^ Iron is encapsulated in hemoglobin cells in the blood, and a small portion of iron in the body binds to iron storage proteins such as ferritin and transferrin.^89^, ^90^ Iron enters the brain mainly through the BBB.^91^

In vitro inflammatory conditions induce microglial polarization and activation.^92^ During neuroinflammation, microglia are activated and polarized into the pro‐inflammatory M1 phenotype and the anti‐inflammatory M2 phenotype in vitro.^92^ A previous study showed that M1 microglia secrete pro‐inflammatory cytokines and nitric oxide, whereas M2 phenotype microglia produce anti‐inflammatory cytokines such as IL‐4, and IL‐13.^92^ Owing to the functional diversity of microglia, microglia are classified by M1, M2a, M2b, and M2c classifications,^93^, ^94^, ^95^ and M1/M2 polarization states could be identified in in vitro conditions.^96^ Previous in vivo studies have shown that the M2 microglia population contributes to the neuroprotective response after stroke,^97^, ^98^ whereas M1 microglial populations aggravate the neuronal damage after stroke.^97^

Since it is difficult to specifically detect M1 and M2 microglia phenotypes in the brain, some studies have suggested that polarization can be estimated using several surface markers, including classical M1 markers CD11b,^99^ CD16,^100^ CD32^98^ and CD86,^101^ and the classical M2 marker CD206.^102^

Microglial activation is very important for controlling neuroinflammatory responses and is directly related to iron uptake.^103^ Under inflammatory conditions, the increased production of pro‐inflammatory mediators increases the uptake of non‐transferrin‐bound serum iron (NTBI) as well as ferritin storage by upregulating both DMT1 and ferritin.^82^ In the inflammatory state, various proteins related to iron metabolism, such as DMT1 and ferritin, contribute to microglial polarization and microglia function.^82^ Within the NTBI uptake pathway in cells, Fe^3+^ is reduced at the cell surface to Fe^2+^ through an endogenous ferrireductase and conveyed into the cytosol through DMT1. During the transferrin‐bound iron (TBI) uptake pathway, iron is mainly combined with transferrin as Fe^3+^ and subsequently enters endosomes through endocytosis.^82^ Microglia can bind to NTBI and TBI as iron forms in the CSF.^104^ In addition, previous studies in rats have shown a positive correlation between microglia polarization and the amount of microglial iron uptake.^82^, ^103^ Furthermore, a recent study reported that knockdown of the ferritin 3 heavy chain homolog (Fer3HCH) leads to mitochondrial dysfunction by decreasing mitochondrial respiration.^105^

Iron deficiency has been reported to cause abnormal brain development and dysfunctions in cognition, motor function, and social behavior patterns.^81^ Excessive accumulation of iron in the brain damages AD‐related brain regions, leading to neuronal loss in the frontal, parietal and hippocampal areas.^106^, ^107^, ^108^, ^109^ The high accumulation of iron in microglia in the cortex and hippocampus of AD patients means that these microglia could be used as a monitoring marker for AD.^108^ Iron chelators could also be used as treatment options for AD based on clinical trial data. Indeed, the iron‐chelating drug deferoxamine has been shown to reduce amyloid plaque formation, and prevent memory loss.^109^, ^110^

Other studies have demonstrated that cerebral iron overload is directly linked to the development of neurodegenerative diseases, such as dementia,^111^ by boosting mitochondrial dysfunction and microglial activation.^112^ In addition, several studies have suggested that iron directly regulates AD neuropathology by boosting amyloid beta peptide aggregation and amyloid beta plaque accumulation, thereby leading to cognitive decline.^113^, ^114^ Iron deficiency aggravates mitochondrial function and oxidative stress, whereas iron overload results in oxidative stress.^115^, ^116^ It has also been suggested that lipid peroxidation, a typical feature of ferroptosis, is an early step in the development of AD.^117^

A recent study using the APP/PS1 AD mouse model demonstrated that excessive iron accumulation in microglia triggers brain dysfunction and changes in brain metabolism.^118^ Subsequently, another study suggested that iron dysregulation is the principal factor behind AD neuropathology and needs to be addressed in order to find a cure for the disease.^119^

These findings show that imbalances in iron levels in the brain contribute to diverse cellular signaling pathways and can aggravate AD neuropathology. Combined, they suggest that the specific mechanisms underlying the link between iron accumulation and brain functions need to be identified in order to find therapeutic solutions for dementia.

## 4 LCN2 AND IRON HOMEOSTASIS

Lipocalin 2, an acute‐inflammatory phase‐related mediator, is quickly secreted in response to inflammatory stimulation.^15^ LCN2 plays a crucial role in various cellular responses, such as in the defense against bacterial infections through the regulation of iron accumulation in the cell, inflammatory signaling, and apoptotic signaling.^120^ LCN2 stimulates glia such as astrocytes and microglia and regulates their production of anti‐ and pro‐inflammatory cytokines under inflammatory conditions via its involvement in iron accumulation.^16^, ^121^ LCN2 is emerging as a promising player in the search for novel dementia treatments because it can regulate inflammatory cytokines and iron accumulation in the CNS.^19^, ^122^

Previous studies have found that LCN2 knockout mice show iron dysregulation in both the peripheral system and the CNS, subsequently leading to synaptic dysfunction and impaired neurogenesis.^22^, ^123^, ^124^, ^125^ A recent study reported that LCN2 could regulate neurogenesis and spine density in hippocampal neurons, as well as neuronal connectivity by modulating iron loading.^124^ Ferreira et al^124^ showed that LCN2 knockout mice display an increase in neuronal differentiation, which is involved in cognitive function. Another study reported that LCN2‐deficient mice displayed more anxiety and depressive behavior as well as cognitive decline,^125^ and LCN2 has also been shown to mediate iron import into the brain.^51^ Furthermore, Xia et al. reported that LCN2 knockout mice show a high level of iron and a severe oxidative stress compared to normal mice.^126^ Conversely, increases in LCN2 levels in the CSF are positively correlated with iron accumulation in basal ganglia regions and elevated levels of transferrin in the CSF.^127^

Previous studies have also reported that the expression of LCN2 in the brain is positively correlated with excessive iron overload in various brain regions such as the cerebral cortex.^59^, ^128^ LCN2 stimulates ferritin expression and iron storage in astrocytes under amyloid beta toxicity,^66^ and has been reported to mediate the import and export of iron into cells under inflammatory conditions.^129^ In fact, recent studies have found that LCN2 contributes to iron homeostasis to regulate the flow of iron from cells into the circulating system involving hepcidin and ferroportin in the brain,^11^ and that it plays a critical role as an iron transport protein by binding to the LCN2 receptor.^15^ LCN2 deficiency has also been reported to impair iron export in cells.^130^

Taken together, the existing evidence suggests that LCN2 is key to the regulation of excessive iron accumulation and inflammation in the brain of dementia patients. Further investigations into the mechanism(s) underlying the link between iron homeostasis and LCN2 in the brain are needed, as iron imbalance is a critical problem leading to memory loss in patients with dementia.

## 5 LCN2 IN DEMENTIA: FOCUS ON NEUROINFLAMMATION

Neuroinflammation is considered an early diagnostic marker of dementia as it is observed from early to late stages of dementia.^131^, ^132^ Neuroinflammation is involved in the activation of glia such as microglia and astrocytes in dementia‐related brain regions,^133^ as well as in the associated cognitive decline.^134^ One study demonstrated that the LCN2 receptor is highly expressed in microglia, astrocytes, and neurons under inflammatory conditions.^135^ Another study suggested that the LCN2 promoter provides binding sites for the inflammatory NF‐κB pathway and CCAAT/enhancer‐binding protein (C/EBP) during inflammation.^136^

In addition, LCN2 induces the polarization of microglia through the activation of NF‐κB signaling as well as the activation of the signal transducer and transcription 3 (STAT3) pathway.^137^ LCN2 knockout mice display a neuroprotective phenotype, which manifests as reduced neuroinflammation under ischemic stroke conditions.^138^, ^139^ Additionally, LCN2 has been reported to promote the activation of astrocytes, and reactive astrocytes are known to stimulate microglial activation under neuroinflammation.^140^ Importantly, activated astrocytes control the expression of inflammatory mediators, transporters, and neurotransmitters and influence neuronal metabolism.^141^, ^142^, ^143^, ^144^

Lipocalin 2 can induce reactive astrocytes through its neurotoxic properties, and subsequently promote cell death.^144^ LCN2 activates both astrocytes and microglia, and exerts neurotoxic effects in neurons, involving memory functions.^144^ Previous in vitro studies have reported that LCN2 is secreted in cultured astrocytes upon lipopolysaccharide stimulation and that it plays a role as an autocrine server.^145^, ^146^ For example, Lee et al^145^ found that LCN2 secretion in glia aggravates neuronal apoptosis mediated by iron and the bcl2 interacting mediator of cell death (BIM) protein, while also boosting neuronal motility. LCN2 also contributes to the inflammatory response by regulating the phagocytic capacity of bacterial clearance in astrocytes and microglia.^147^, ^148^

To summarize, these findings show that LCN2 induces the activation of microglia and astrocytes, regulates their functions, causes neuronal cell death, and influences neuronal function when in a state of neuroinflammation. Therefore, the modulation of LCN2 levels in the brain may be key to reducing pro‐inflammatory responses that occur in the brain of individuals with dementia.

## 6 LCN2 IN DEMENTIA: FOCUS ON METABOLIC ALTERATIONS

Recent studies have emphasized and investigated the positive correlations between metabolic syndromes such as diabetes, obesity, and dementia.^149^, ^150^ Numerous studies have reported that metabolic changes commonly observed in patients with metabolic syndromes, such as hyperglycemia, dyslipidemia, hypertension, and insulin resistance, are strongly related to the neuropathology of dementia.^151^, ^152^, ^153^ Clinically, diabetic neuropathy, such as memory loss, is a complication of diabetes.^154^, ^155^

Several studies have suggested that LCN2 is linked to inflammatory responses in metabolic disorders, including obesity,^156^ in which the pathway involves NF‐κB,^157^ C/EBP,^158^ and estrogen response elements.^157^, ^159^ Other studies have reported that elevated LCN2 levels are observed in animal models of diabetes and obesity^160^, ^161^ and that LCN2 aggravates insulin resistance as well as lipid metabolism.^162^ Furthermore, blood levels of LCN2 are reported to be positively correlated with total body fat mass and glycated hemoglobin (HbA1c),^163^ as well as with hyperglycemia and insulin resistance in patients with metabolic syndrome.^164^, ^165^, ^166^ Recent studies have also reported that the levels of LCN2 in blood serum, adipose tissue, and liver are increased in models of obesity.^160^, ^167^ In fact, Yan et al^160^ found that LCN2 expression in adipocytes leads to insulin resistance in adipocytes, and that LCN2 could control insulin sensitivity in patients with obesity. Based on this data, the authors suggested that LCN2 likely regulates the secretion of adipokines in adipose tissues, and ultimately controls both the inflammatory state and metabolic balance.^160^

The expression and secretion of LCN2 are higher in mature adipocytes than in preadipocytes^23^ and are induced by various inflammatory cytokines and factors.^168^ Finally, the change of fat mass leads to increased cytokines and adipokines, and subsequently, aggravates the state of inflammation, and increases vascular damage. LCN2 influences vascular remodeling, regulates atherosclerotic plaque formation caused by metabolic changes, and is ultimately involved in the onset of VD.^168^, ^169^, ^170^

To summarize, the current evidence shows that LCN2 is involved in insulin sensitivity, glucose metabolism, vascular homeostasis, and hyperglycemia related to metabolic syndromes. These strong correlations between LCN2 and metabolic factors should be highlighted and considered in the search for therapeutic options for the treatment and prevention of dementia, considering that the onset and development of dementia are strongly related to metabolic disorders, including hyperglycemia and insulin resistance.

## 7 CONCLUSIONS

Here, we emphasize that LCN2 has crucial pathogenic roles in dementia through the regulation of iron homeostasis, neuroinflammation, and insulin resistance. The onset and progression of dementia are influenced by a variety of LCN2‐mediated mechanisms including inflammation, insulin resistance, iron accumulation, immune response, neuronal cell damage, and glia dysfunction.

**FIGURE 1:**
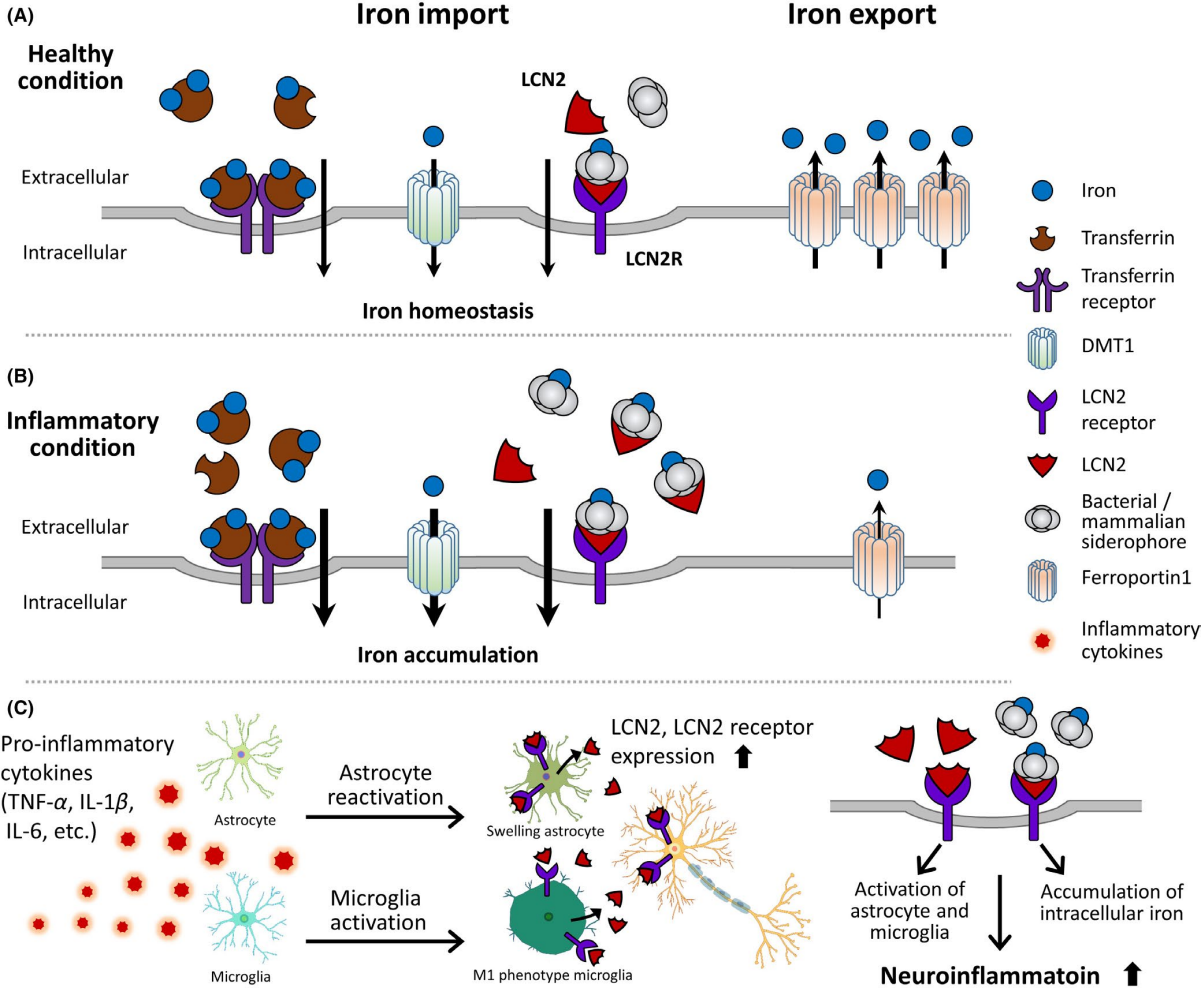
The role of LCN2 in iron accumulation and neuroinflammation. (A,B) LCN2 is related to the import and export of iron into neuronal cells. (B) Under neuroinflammation conditions, LCN2 binds with many bacterial/mammalian siderophores and subsequently increases iron accumulation from the extracellular space into the intracellular space. (C) LCN2 triggers astrocyte reactivation and swelling as well as the induction of M1 microglial phenotype. Finally, LCN2 activates the production of pro‐inflammatory cytokines in astrocytes and microglia, and promotes the accumulation of intracellular iron, leading to the aggravation of neuroinflammation. DMT1, divalent metal transporter 1; IL‐1β, interleukin‐1 beta; IL‐6, interleukin −6; LCN2, lipocalin 2; LCN2R, lipocalin 2 receptor; TNF‐α, tumor necrosis factor‐α

**FIGURE 2:**
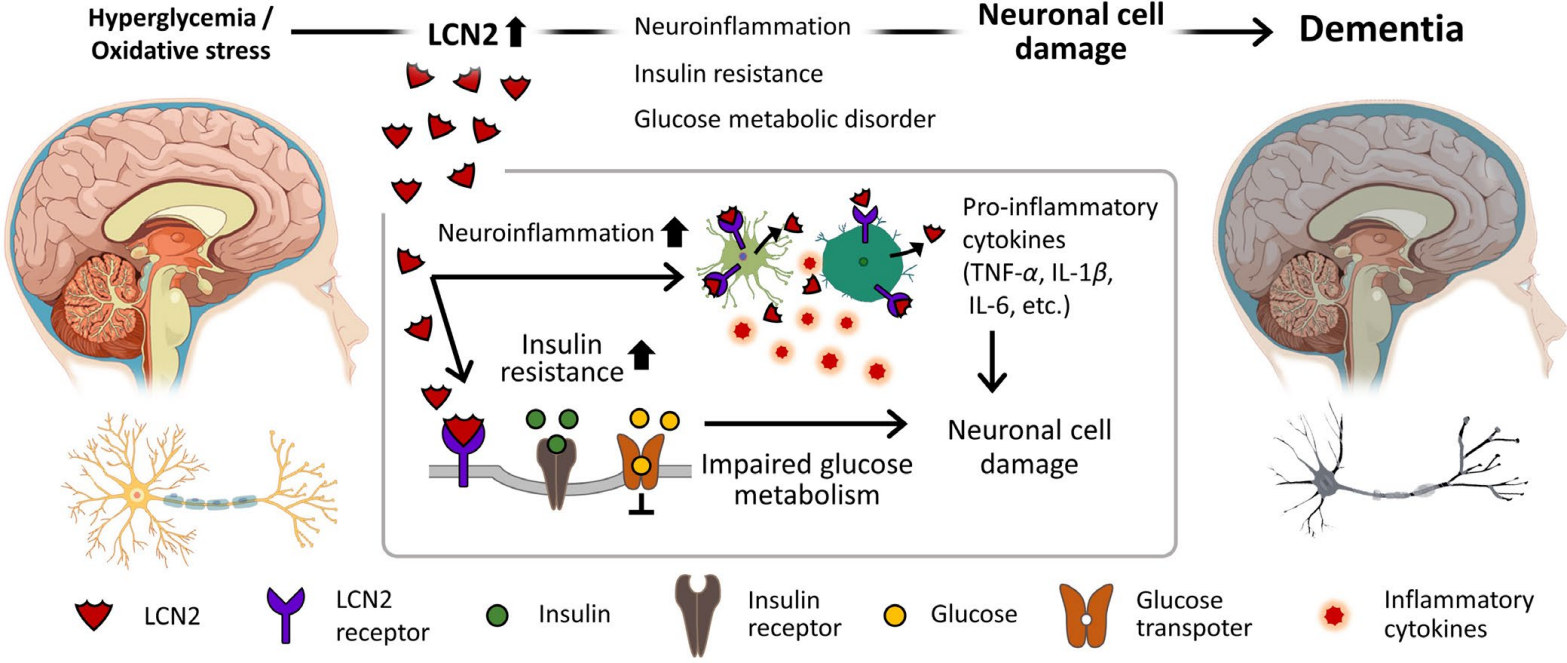
The role of LCN2 in neuronal cell damage under hyperglycemia and oxidative stress. Hyperglycemia and oxidative stress cause cognitive decline in the brains of patients with dementia. Elevated levels of LCN2 contribute to the increase in insulin resistance and the aggravation of neuroinflammation in the brain. Ultimately, these responses result in neuronal cell death. IL‐1β, interleukin‐1 beta; IL‐6, interleukin −6; LCN2, lipocalin 2; TNF‐α, tumor necrosis factor‐α

Given the significant evidence supporting the involvement of LCN2 in the demented brain, we believe that the level of LCN2 in the brain is a critical factor in the regulation of risk factors for dementia. Thus, we reviewed the roles of LCN2 in dementia and came to the following three conclusions: first, LCN2 contributes to excessive iron accumulation and is ultimately involved in the neuropathology of dementia (Figure 1A,B), suggesting that LCN2 influences neuronal cell apoptosis by regulating iron accumulation in the brain. Second, LCN2 accelerates neuroinflammation by regulating the activation and function of glia and subsequently aggravates the induced neuroinflammation by regulating NF‐kB signaling and the STAT3 pathway. Appropriate modulation of LCN2 may enhance the neuropathology of dementia (Figure 1C). Finally, LCN2 can control metabolic homeostasis, including insulin sensitivity, hyperglycemia, and dyslipidemia through the modulation of NF‐κB, C/EBP signaling, HbA1c level, and body fat mass (Figure 2).

The modulation of LCN2 levels may contribute to the attenuation of the neuropathology of dementia, given that recent research focused on the chemotherapy in CNS disease including dementia as well as brain tumor.^171^ The immunotherapy using LCN2 neutralization may be a promising clinical approach to treat and prevent neuropathology in dementia, suggesting that LCN2 deficiency markedly suppresses neuroinflammation based on preclinical studies for various CNS disorders,^172^ such as traumatic brain damage,^173^ experimental autoimmune encephalomyelitis,^174^ and stroke.^175^ Thus, we emphasize that LCN2 may be a critical target for the treatment and prevention of dementia.

## CONFLICT OF INTEREST

The authors have no conflicts of interest to declare.

## AUTHOR CONTRIBUTIONS

Daejin Lim and Jae‐ho Jeong and Juhyun Song contributed to the writing of text and provided the table and figures. Juhyun Song wrote, revised, and finalized the whole manuscript. All authors have read and approved the final manuscript.

## Data Availability

Data sharing not applicable to this article as no datasets were generated or analyzed during the current study.
